# A Novel Missense Variant in *LHX4* in Three Children with Multiple Pituitary Hormone Deficiency Belonging to Two Unrelated Families and Contribution of Additional *GLI2* and *IGFR1* Variant

**DOI:** 10.3390/children12030364

**Published:** 2025-03-14

**Authors:** Claudia Santoro, Francesca Aiello, Antonella Farina, Emanuele Miraglia del Giudice, Filomena Pascarella, Maria Rosaria Licenziati, Nicola Improda, Giulio Piluso, Annalaura Torella, Francesca Del Vecchio Blanco, Mario Cirillo, Vincenzo Nigro, Anna Grandone

**Affiliations:** 1 Department of Child, Woman, General and Specialized Surgery, University of Campania “L. Vanvitelli”, L. De Crecchio 4 Street, 80138 Naples, Italy; claudia.santoro@unicampania.it (C.S.); emanuele.miragliadelgiudice@unicampania.it (E.M.d.G.); anna.grandone@unicampania.it (A.G.); 2 Department of Precision Medicine, University of Campania “Luigi Vanvitelli”, Sant’Andrea delle Dame Square L. De Crecchio 7 Street, 80138 Naples, Italy; antonella.farina1@unicampania.it (A.F.); giulio.piluso@unicampania.it (G.P.); annalaura.torella@unicampania.it (A.T.); francesca.delvecchioblanco@unicampania.it (F.D.V.B.); vincenzo.nigro@unicampania.it (V.N.); 3 Pediatric Endocrinology Unit, Sant’Anna e San Sebastiano Hospital, Palasciano Street, 81100 Caserta, Italy; 4 Neuro-Endocrine Diseases and Obesity Unit, Department of Neurosciences, Santobono-Pausilipon Children’s Hospital, Via Egiziaca a Forcella, 18, 80139 Naples, Italy; mr.licenziati@santobonopausilipon.it (M.R.L.); n.improda@santobonopausilipon.it (N.I.); 5 Advanced MRI Neuroimaging Centre, Department of Advanced Medical and Surgical Sciences, University of Campania “Luigi Vanvitelli”, Sant’Andrea delle Dame Square, 80138 Naples, Italy; mario.cirillo@unicampania.it; 6 MRI Research Center SUN-FISM, University of Campania “Luigi Vanvitelli”, Sant’Andrea delle Dame Square, 80138 Naples, Italy; 7 Telethon Institute of Genetics and Medicine (TIGEM), Via Campi Flegrei 34, 80078 Pozzuoli, Italy

**Keywords:** combined pituitary hormone deficiency, congenital hypopituitarism, whole exome sequencing, pituitary stalk interruption syndrome, *LHX4*, *GLI2*, *IGF1R*, pituitary

## Abstract

Background: Multiple genes can disrupt hypothalamic–pituitary axis development, causing multiple pituitary hormone deficiencies (MPHD). Despite advances in next-generation sequencing (NGS) identifying over 30 key genes, 85% of cases remain unsolved, indicating complex genotype–phenotype correlations and variable inheritance patterns. Objective: This study aimed to identify the MPHD genetics in three probands from two unrelated families. Methods: Family A had one affected child, while Family B had two affected siblings. All probands exhibited poor growth since birth, and family B’s probands were born small for gestational age. Growth hormone deficiency was confirmed in all subjects. Family B’s probands responded poorly to growth hormone treatment compared to the first patient. Furthermore, Family A’s proband and Family B’s younger sibling developed central hypothyroidism, while Family B’s older sibling presented hypogonadotropic hypogonadism. Brain magnetic resonance imaging (MRI) revealed pituitary hypoplasia, ectopic posterior pituitary gland, and small sella turcica in all probands. Patients and their available relatives underwent NGS. Results: NGS identified the same novel and likely pathogenic LHX4 variant (c.481C>G) in all probands despite the families being unrelated. Additionally, Family A’s proband carried a *GLI2* variant (c.2105C>A), and Family B’s probands carried an IGF1R variant (c.166G>A), both interpreted as being of uncertain significance. Conclusions: This study confirms that heterozygous pathogenic variants of LHX4 can cause MPHD associated with a specific neuroradiological triad of abnormalities despite incomplete penetrance and variable phenotype. Moreover, the co-occurrence of the other two gene variants was debated. The *IGF1R* variant could explain the unusually poor response to growth hormone therapy in Family B, suggesting an oligogenic mechanism underlying the phenotype.

## 1. Introduction

Somatic growth is a complex process of evolutionary age resulting from continuous crosstalk between genetic assets and environmental factors. Evaluating the genetic background of patients affected by growth deficiency can be extremely challenging as genetic causes have been identified only in a small number of cases. Genetic variants in genes involved in growth plate development or, less frequently, in growth hormone secretion and action are usually found in genetic forms of short stature. Congenital growth hormone deficiency is a rare condition with a reported incidence of 1 out of 4000 live births. It can be due solely to growth hormone deficiency or become clinically apparent in combination with other deficiencies, a condition better known as multiple pituitary hormone deficiency (MPHD) [1,2]. Genetic variants, antenatal insults, or a combination of both can alter correct pituitary development and its functioning. However, only 5–10% of the cases of MPHD can be explained by a monogenic defect, suggesting a more complex pathogenesis [3,4,5,6,7].

MPHD is characterized by the reduced synthesis of at least two pituitary hormones (IGF1, TSH, ACTH, LH, FSH, PRL). Hormone deficiencies can develop simultaneously in the first years of life or can develop gradually over time. The clinical manifestations of the disorder may vary due to variability in the disease genes involved and/or the influence of environmental factors during pituitary organogenesis. GH deficiency is usually the first hormonal alteration detected in these patients. Poor growth can be detected early in life, manifesting from 2 to 8 years of age, and a clinical history of cryptorchidism and hypoglycemia at birth can be present [8].

MPHD term encompasses many heterogeneous phenotypes that can be divided into two main groups: patients manifesting extra-pituitary abnormalities or malformations on MRI (such as pituitary stalk interruption syndrome or midline defects) and those presenting a “pure” endocrine phenotype, including anterior pituitary hormone deficiencies (progressive or not), showing no alterations in hypothalamus–pituitary morphology at MRI [9]. These phenotypes are usually due to variants of late-acting pituitary-specific transcription factors. In such context, *PROP1* gene variants remain the most frequently reported genetic defect [3]. Multiple disease-causing genes can disrupt hypothalamic–pituitary axis ontogeny (or embryonic development), leading to MPHD in humans [10,11,12]. The list is becoming longer thanks to the massive use of next-generation sequencing (NGS) and includes at least 30 genes encoding transcription factors involved in pituitary development. There are several mechanisms of inheritance, which include an oligogenic pattern and autosomal dominant inheritance with incomplete penetrance and variable phenotype [13,14,15,16].

Here, we report a novel, likely pathogenic, *LHX4* variant identified by exome sequencing in three patients belonging to two apparently unrelated pedigrees. We also discussed two missense variants in *GLI2* and *IGF1R*, which co-occurred in the affected patients of the two families.

## 2. Materials and Methods

### 2.1. Exome Sequencing

Genomic DNA (gDNA) was extracted from peripheral blood using standard procedures with FlexiGene DNA Kit (Qiagen, Hilden, Germany); purity analysis of obtained gDNA via spectrophotometer (Nanodrop ND 1000, Thermo Scientific, Waltham, MA, USA) was performed. Its quality was verified by agarose gel electrophoresis, and its concentration was measured using Qubit Fluorometers (ThermoFisher Scientific, Waltham, MA, USA). Each proband and their parents (family trio) were analyzed by whole exome sequencing (WES). For library preparation, we used SureSelect QXT Human All Exon V7 kits compatible with the Illumina platform (Agilent Technologies, Santa Clara, CA, USA), following the manufacturer’s instructions.

Purified DNA quality control was performed by a High Sensitivity DNA kit and TapeStation Analysis Software v3.2 (Agilent Technologies). The libraries were sequenced using a NovaSeq6000 system (Illumina, San Diego, CA, USA) by performing paired-end runs covering at least 2 × 150 nt. A medium coverage of 100× is usually obtained, with 90% of the regions covered by at least 40 reads. Generated sequences, aligned against the hg19 genome assembly, were analyzed using an in-house pipeline designed to automate the analysis workflow. The classification of the variants’ pathogenicity was performed according to the American College of Medical Genetics (ACMG) guidelines [17]. All pathogenic variants detected were confirmed by direct Sanger Sequencing with BigDye v3.1 sequencing kit (Applied Biosystems, Waltham, MA, USA) in a 3500 Genetic Analyzer (Applied Biosystems).

### 2.2. Patients

**Figure 1:**
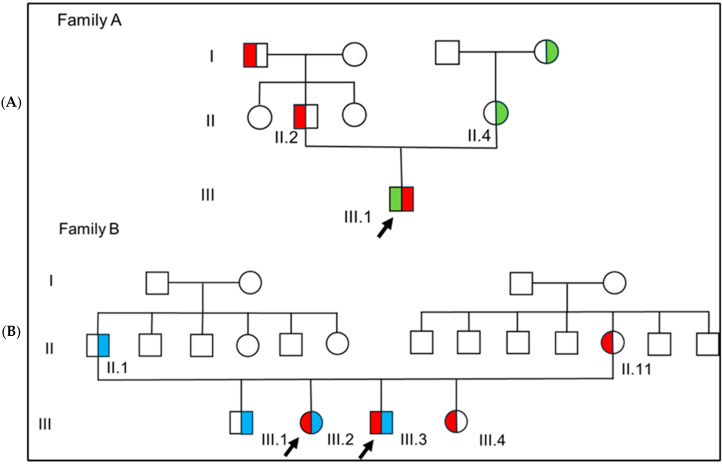
Pedigree charts for family A (**A**) and family B (**B**). Color chart: red for heterozygous state for *LHX4* (c.481C>G), green for *GLI2* variant (c.2105C>A), and blue for *IGF1R* variant (c.166G>A); arrows indicate probands.

We conducted a review of clinical, radiological, and genetic findings of three patients, one male and two siblings, with a phenotype consistent with MPHD (see Figure 1).

**Figure 2:**
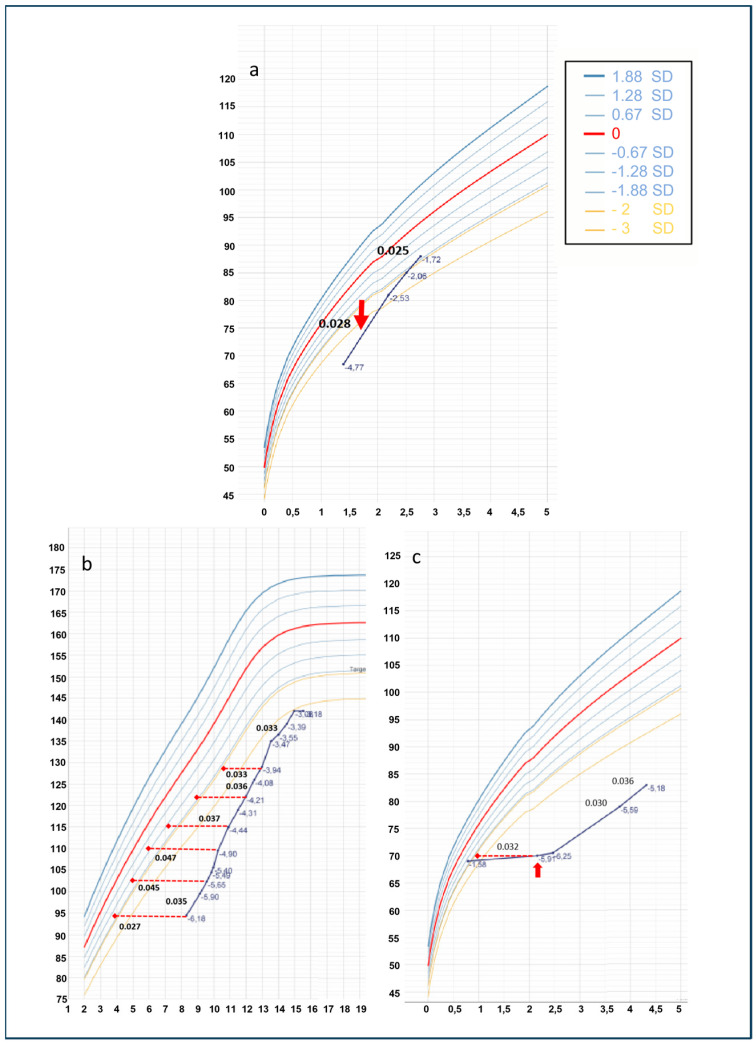
Growth charts of probands: A.III.1 (**a**), B.III.2 (**b**), and B.III.3 (**c**). Numbers on the right express changes in mean rGH dosage as mg/kg/day. Continuous black line represents patient linear growth upon rGH therapy. Red dashed lines report height for bone age. Red arrows point to the time of rGh therapy onset.

Weight, height, and head circumference (HC) standard deviations (SDs) at birth were calculated using the Bertino growth chart for Italian newborns in 2010 [18]. WHO Growth Chart 2006 for sex and age were used to calculate auxological parameters SDs for childhood [19]. Growth curves (Figure 2) were built with the Growth 4 SIEDP calculator and partially modified to include height corrected for bone age and administered recombinant growth hormone (rGH) mean dose. Clinical and neuroradiological examinations were performed on all probands and their carrier parents. The cut-off value for the GH-stimulation test has been set at 8 ng/mL according to the Italian Society of Endocrinology and Diabetology indications and the guidelines issued by the Italian Medicines Agency (AIFA) regarding the prescription of growth hormone and its analogs (somatropin, somatrogon) under the National Health Service (AIFA Note 39, G.U. 154/2014) [20].

The patients’ parents gave written informed consent for publication in accordance with the Declaration of Helsinki. Clinical and neuroradiological examinations were performed for all probands and their carrier relatives.

### 2.3. Family A

The proband (Figure 1, A.III.1) was the only child of two non-consanguineous healthy parents. Mid-parental height was 168.7 cm (−1.16 SD).

He was born full-term by vaginal delivery with a birth weight of 3300 gr (−0.47 SD), length of 49 cm (−0.11 SD), and HC of 33 cm (−0.82 SD). At birth, bilateral cryptorchidism was diagnosed. At 13 months of age, growth deceleration became clinically apparent, and the first hormone assessment revealed undetectable IGF1 levels, <15 ng/mL (normal reference value for age and sex, r.v.: 14–203 ng/mL), and low levels for age of basal GH (0.36 ng/mL, r.v.: 2–10 ng/mL). The patient showed no defects in thyroid or adrenal functions.

**Figure 3:**
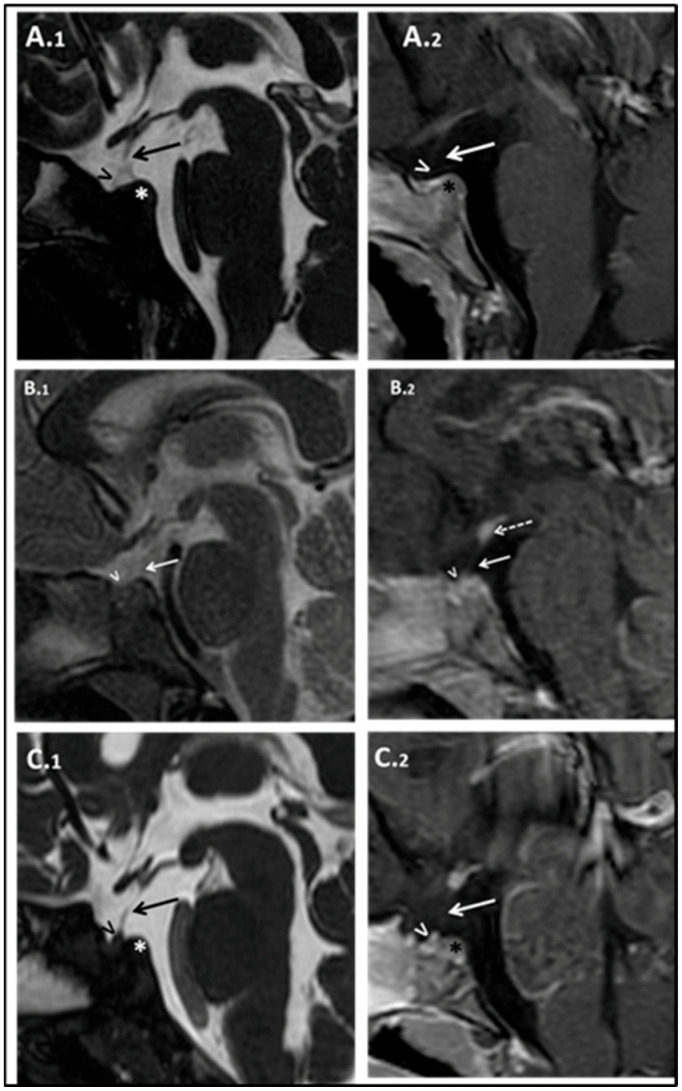
Patient’s brain MRI studies. A.III.1’s Sagittal MRI images T2 weighted (**A.1**) and T1 weighted with gadolinium (**A.2**). Subject B.III.2. Sagittal MRI images T2 weighted (**B.1**), and T1 weighted with gadolinium (**B.2**). B.III.3 Brain MRI. Sagittal MRI images T2 weighted (**C.1**) and T1 weighted with gadolinium (**C.2**). Hypoplastic anterior pituitary lobe (indicated by “>”), small poorly developed sella turcica (indicated by “*”), absent pituitary stalk (see the “arrow”), and ectopic posterior pituitary located at the level of the median eminence.

At 17 months of age, his length was 68.5 cm (−2 SD), and his weight was 8.17 Kg (−2 SD); the HC was normal. No facial dysmorphisms were present. Psychomotor development was normal. IGF1 was confirmed lower than normal: 7.1 ng/mL (−2.31 SD). According to the diagnostic guidelines for GH deficiency in children under 2 years of age, a brain MRI was suggested, yet parents initially refused the sedation procedure needed to perform the exam. Thus, we performed an arginine stimulation test that confirmed the suspicion of GH deficiency: peak GH of 1.18 ng/mL. Other pituitary hormones were still within the normal range (Supplementary Materials, Table S1). Subsequently, parents agreed to perform the brain MRI that documented poorly developed sella turcica, marked anterior pituitary hypoplasia, pituitary stalk interruption, and ectopic posterior pituitary (Figure 3A.1,A.2).

The patient started recombinant GH (rGH) therapy at the age of 1.7 years with a starting dose of 0.028 mg/kg/day. He presented a brilliant response at the 12th month of therapy and thereafter a complete normalization of the height that resulted within the genetic target (see Figure 2a). During the treatment, at 21 months of age, central hypothyroidism (TSH 1.15 mcUI/mL with an FT4 0.65 ng/dL) became evident.

### 2.4. Family B

Patients B.III.2 and B.III.3 are two siblings of unrelated parents (Figure 1, B.III.2, and B.III.3). Family history was consistent for short stature in maternal pedigree: the mother herself was 154 cm tall (−1.47 SDS) while grand-mother and grand-mother’s sister were both 150 cm tall (−2.12 SDS). A healthy older brother aged 21, B.III.1, manifested reduced final height (159.4 cm; −2.5 SDS) within the genetic target.

Both patients had received a diagnosis of GH deficiency at the age of 8.75 years and 2.5 years, respectively. Due to the poor response to rGH replacement therapy, they were referred for a level III endocrinological evaluation to retrieve further investigations. Patient B.III.2 was born full-term by spontaneous delivery with a birth weight of 2500 gr (−1.41 SD), length of 45 cm (−1.83 SD), and HC of 33.8 cm (0.1 SD). Growth curve analysis did not show the physiological postnatal catch-up growth, with weight and length persistently below the 3rd percentile. At the first endocrinological evaluation, at the age of 8.3 years, her height was 94.5 cm (−6.18 SD), remarkably below mid-parental height (151 cm; −1.97 SD), with a weight of 15 kg (−4.08 SD). Left wrist and hand X-ray bone age was 4.7 years, remarkably delayed (according to the RUS method).

Complete hormonal screening revealed low levels of IGF1 (21.23 ng/mL; −2.48 SD) (see Supplementary Materials, Table S2). A growth hormone stimulation test confirmed severe GH deficiency (GH peak after arginine and GHRH stimulation of 5.8 ng/mL and 2.7 ng/mL, respectively). Brain MRI imaging evidenced the triad, hypoplastic anterior pituitary gland, thin pituitary stalk, and ectopic posterior pituitary gland, along with poorly developed sella turcica (see Figure 3B.1,B.2).

She started rGH therapy (with an initial dose of 0.027 mg/kg/day). Due to poor clinical response despite good patient compliance to therapy, the mean rGH dose has been rapidly increased to 0.045 mg/kg/day with only a marginal improvement in height growth (see Figure 2b).

Puberty that spontaneously started at 10 years of age stopped at 13.6 years, configuring primary amenorrhea with a hormonal profile suggesting hypogonadotropic hypogonadism (LH 9.6 mUI/mL, FSH 7.7 mUI/mL with low 17beta-estradiol 18.5 pg/mL, r.v. for pubertal stage IV: 15–85 pg/mL). Hormone replacement therapy with estro-progesterone started at the age of 13.8 years old. No further pituitary deficiencies were observed to date.

Patient B.III.3 was born at term with a weight of 2200 gr (−2.09 SD), a length of 38 cm (−4.28 SD), and an HC of 32 cm (−1.56 SD). Similarly to the sister, he did not present postnatal catch-up growth, so at 9 months, weight and length were already below the 3rd centile, and he was first referred to a pediatric endocrinologist. At 16 months of age, he received a diagnosis of GH deficiency due to a persistent low level of IGF1 (14.7 ng/mL; −1.83 SD, r.v.: 14–203 ng/mL) during symptomatic hypoglycemia. At that time, he was 70 cm tall (−3.96 SD), below his mid-parental height (164 cm; −2.14 SD); his weight was 7.520 kg (−3.04 SD), and his BMI was 15.35 kg/m^2^ (−0.80 SD). A brain MRI scan depicted hypoplastic anterior pituitary gland, thin pituitary stalk, and ectopic posterior pituitary gland (see Figure 3C.1,C.2). Moreover, poorly developed sella turcica and olfactory bulbs and tract hypoplasia–agenesia were retrieved.

At 2.5 years of age, he started rGH treatment at a mean dose of 0.027 mg/kg/day with a very poor growth response, similar to his sister; even in this case, dose adjustment to higher mean dosage (0.036 mg/kg/day) failed to significantly increase treatment response (Figure 2c).

At the age of 4.7 years old, when the patient came to our service, his height was 88.4 cm (−4.4 SD), and his BMI was normal (BMI 14.5 kg/m^2^, −0.61 SD) as long as his HC (49 cm, −1.29 SD). He presented hypotelorism and thin uvula; dysmorphisms were also present in his mother.

He developed central hypothyroidism one year later (see Supplementary Materials, Table S3).

## 3. Results

WES analysis revealed that all probands (A.III.1, B.III.2, and B.III.3) carried the same *LHX4* variant c.481C>G; p.Arg161Gly, not yet reported in any variant database and interpreted as pathogenic according to ACMG criteria [17] (see Supplementary Materials, Table S4).

Varsome analysis resulted in an in silico predictor metascore of 16.

The rare *LHX4* variant, c.481C>G; p.Arg161Gly, was paternally inherited in the proband belonging to family A, whereas the mother was the carrier in family B.

**Figure 4:**
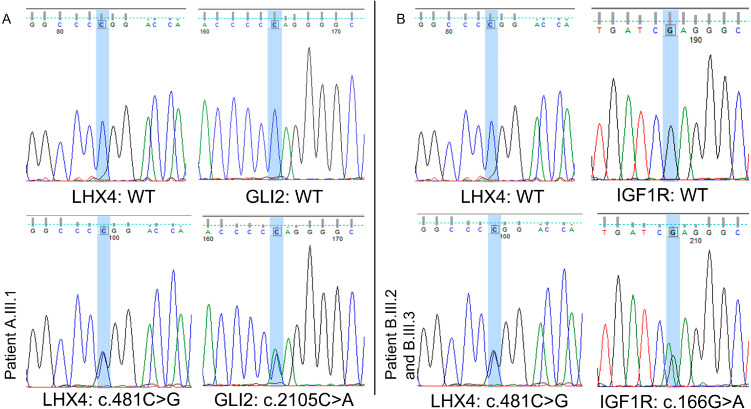
Sanger sequencing chromatogram reporting wild-type gene sequence and all variants in family A (panel (**A**)) and B (panel (**B**)). Each colored line refers to a specific nucleotide (black for guanosine, green for adenine, dark blue for cytosine, and red for thymine), while light blue columns highlight the specific sites for nucleotide changes.

Two additional variants in two different genes potentially related to the poor growth were detected in the two families (Figure 4 and Supplementary Materials Table S4, for further details). A segregation study of all variants was performed, and endocrinological and neuroradiological evaluations of all carriers and their available relatives were carried out (please see Table 1).

Patient A.III.1 had a maternally inherited GLI2 variant: c.2105C>A; p.Pro702Gln classified as a VUS and with a very low frequency in gnomAD Exomes database (0.00000805) (see Supplementary Materials, Table S4).

WES analysis was extended to the proband’s grandparents, and the inheritance path was reconstructed in the pedigree (Figure 1).

In family B, both affected siblings presented the *IGF1R* variant (c.166G>A; p.Glu56Lys), which has never been reported in a public database and is similarly classified as a VUS.

A segregation study of all variants was performed since endocrinological and neuroradiological evaluations of all carriers and relatives were available.

Regarding the carrier of the *LHX4* variant (the father of patient A.III.1), his brain MRI revealed an anterior pituitary of normal dimensions, but slightly asymmetric with a prevalence of the paramedian left portion due to the presence of a perimillimetric spot; pituitary stalk slightly deviated to the left deviation (see Supplementary Materials, Figure S1). He reached a normal final height of 169.5 cm (−1.01 SD) and a normal head circumference of 55.2 cm (+0.07 SD). Complete hormonal investigation revealed a level of IGF1 in the lower part of normality (133 mcg/L, −0.52 SD), whereas other hormones were within the normal range (LH 6.8 UI/L, r.v.: 1.7–8.6 IU/L; FSH 3.9 UI/L, r.v.:1.5–12.4 IU/L; testosterone 6.08 ng/mL, r.v.: 2.49–8.36 ng/mL; TSH1.13 mUI/L, r.v.: 0.27–4.20 mUI/L; FT4 1.55 ng/dL, r.v. 0.93–1.7 ng/dL; prolactin 114 mUI/mL, r.v.: 86–324 mUI/mL, cortisol 8.6 µg/dL, r.v. 4.5–24 µg/dL).

The mother of the siblings belonging to family B, B.II.11, showed normal final height and head circumference (height 154 cm; −1.41 SD; HC 55 cm; +0.63 SD). She presented hypotelorism and thin uvula. She had no endocrinological deficiency. However, her brain MRI, requested based on the genetic results, uncovered pituitary gland size at the lower range of normality for age and sex, with a thin distal two-thirds pituitary stalk (see Supplementary Materials, Figure S2).

**Figure 5:**
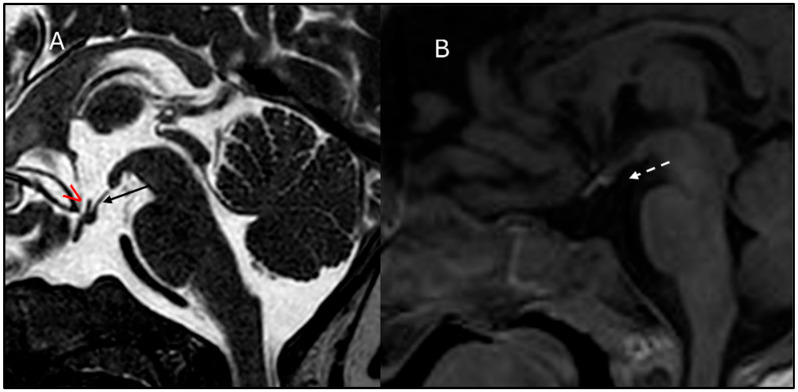
Family B, III.4 Brain MRI. Sagittal MR images T2 weighted (**A**) and T1 weighted (**B**) Pituitary morphology: Hypoplastic anterior pituitary lobe (>), thin pituitary stalk (arrow), and ectopic posterior lobe located at the level of the median eminence (dashed arrow).

Patient B.III.4, the youngest sibling, was born at full term, adequate for gestational age, a few months after the genetic diagnosis of her two siblings. She resulted positive in the screening for congenital hypothyroidism and was diagnosed with central hypothyroidism, along with central hypercortisolism and undetectable gonadotropin levels during her mini puberty, suggesting hypogonadotropic hypogonadism (see Supplementary Table S5 for further details). The child started thyroid hormone replacement therapy and hydrocortisone at 20 days of life. At 4 months of age, she weighed 5.464 kg (−1.55 SDS), whereas her length was 56 cm (−3.86 SD). Relative macrocephaly was present (HC 41 cm; 0 SD). At the clinical examination, she presented a domed forehead and slightly deep-set eyes. Neurodevelopment was normal. Brain MRI showed posterior pituitary gland ectopy, thin pituitary stalk, and anterior pituitary hypoplasia (Figure 5).

Intriguingly, patient B.III.4 was found to carry only the *LHX4* variant, not the *IGF1R* variant.

Regarding the carriers of *GLI2* and *IGF1R* variants, subject A.II.4 (the mother of patient A.III.1) and subject B.III.1 (older brother of the family B probands), respectively, were further investigated.

Subject A.II.4 underwent a brain MRI, which depicted normal hypothalamus–pituitary region morphology (see Supplementary Materials, Figure S3). The pituitary hormonal function was preserved.

Subject B.III.1, carrying only the paternal *IGF1R* variant (c.166G>A; p.Glu56Lys), was born at term, small for gestational age: weight at birth was 2520 gr (−1.44 SD); length was 45 cm (−2 SD) and CC 32.5 cm (−1.21 SD). At the time of examination, at the age of 21 years, he was 159.5 cm tall (−2.5 SD); he weighed 64.5 kg (−0.45 SD); his BMI was 25.36 kg/m^2^ (0.7 SD). Pubertal stage was PH5G5. Laboratory test revealed a preserved pituitary function: IGF-1 352 ng/mL (r.v.: 91–442 ng/mL); LH 3.3 mU/mL (r.v.: 1.3–9.6); FSH 2.5 mUI/mL (r.v.: 1.2–15.8); free testosterone 18.72 nmol/L (r.v.: 5.25–20.7); TSH 1.15 uU/mL (r.v.: 0.5–4.2); FT4 0.85 ng/dl (r.v.: 0.9–1.7); PRL 15.9 ng/mL (r.v.: 4–23); cortisol 12 µg/dl (r.v.: 7–25).

Subject B.II.1, the father of the probands B.III.2 and B.III.3, carrying the same *IGF1R* variant, presented short stature (H 162.3 cm; −2.32 SD). Unfortunately, he was not available for further endocrinological investigation.

## 4. Discussion

We report on three patients belonging to two different kindreds with the MPHD in whom the application of WES revealed a combination of a novel *LHX4* variant, c. 481C>G (p.Arg161Gly), with a very rare variant in *GLI2* (c.2105C>A; p.Pro702Gln) in patient A.III.1 and a novel variant in *IGFR1* (c.166G>A; p.Glu56Lys) in patients B.III.2 and B.III.3.

The *LHX4* gene is a member of the LIM-homeodomain family of transcription factors required for normal development of the pituitary gland. It is located in chromosomal position 1q25.2. Its main isoform is made by six exons encoding a 390 amino acids protein. *LHX4* is expressed in Rathke’s primordial pouch and, in adulthood, in the anterior and intermediate lobes [21]. The LHX4 protein acts as a transcriptional regulator during pituitary gland and nervous system development [22].

In humans, *LHX4* is a rare cause of MPHD, explaining only 1.4% of all cases [23]. Its heterozygous pathogenic variants are responsible for Pituitary Hormone Deficiency, Combined, 4, (CPHD4, MIM 262700), usually associated with pituitary stalk interruption syndrome (PSIS), a peculiar neuroradiological pattern due to an abnormal embryogenesis of the pituitary gland [24]. High clinical variability, even in the same family, is described for CPHD4. Both heterozygous missense and nonsense variants have been reported (Supplementary Table S6), causing an incomplete penetrance and a variable MPHD, which is thought to be secondary to haploinsufficiency rather than dominant-negative effects [25,26]. The homozygous status of the variant p.T126M in *LHX4* has been reported in a congenital lethal form of hypopituitarism in three relatives who died within the first week of life [27].

The novel missense variant here reported in *LHX4*, c.481C>G; p.Arg161Gly, occurred in two apparently unrelated families, which, however, belong to the same geographic region, suggesting the chance of ancestry effect. Under the neuroradiological point of view, only one of our three probands (A.III.1) showed a complete neuroradiological PSIS phenotype, while the other three patients from family B, with MPHD deficiency related to *LHX4,* presented posterior pituitary gland ectopy, poorly developed sella turcica, anterior pituitary hypoplasia, and a thinner pituitary stalk. The *LHX4* parent carriers showed slightly normal pituitary neuroradiological findings.

The *LHX4* variant is not reported in Gnomad and is classified as likely pathogenic according to ACGM. A pathogenic missense variant that occurs two codons downstream from the one of our interest (c.487A>C, p.Thr163Pro) is reported in the literature [23]. The latter, as in our case, falls within the homeodomain, potentially influencing gene expression. We also analyzed the alignment between different species of the *LHX4* amino acid sequence and observed that the R (arginine) at position 161 is conserved among vertebrates and other species like Drosophila. A high degree of conservation of this residue strongly suggests that an amino acid substitution at this position can alter protein function and be responsible for pathological conditions.

The c.481C>G variant, here described, led to a phenotype that fully recapitulates the one originally reported by Machinis et al. in 2001 [28]: the defect was inherited in an autosomal dominant manner, with incomplete penetrance and variability regarding clinical expression.

On brain MRI, all probands presented anterior pituitary hypoplasia and poorly developed sella turcica, as originally described by Machinis et al. 2001 [28]. In addition, posterior pituitary ectopia was present, which was identified as an inconstant phenotypic trait in the previous literature [25,29]. It is worth noting that brain MRI performed in carriers parents, A.II.2 and B.II.1, revealed less severe brain abnormalities. These findings were in line with the literature showing how patients bearing *LHX4* variants can have highly variable neuroradiological features ranging between completely normal imaging and a PSIS triad, even within the same family [25,26].

The genotype and clinico-radiological phenotype of all patients with *LHX4* variants reported today were summarized in Figure S4 and Table S6 of Supplementary Materials, respectively [7,23,24,25,26,28,29,30,31,32,33,34,35,36,37,38,39]. No evident genotype–phenotype correlations exist, and the phenotype is highly heterogeneous. Among the 54 patients harboring an *LHX4* variant described in the literature, 45 patients underwent a brain MRI study. The typical triad of PSIS was documented in 13.3% (6/45) of them. This frequency became higher at 35.6% (16/45) when patients with anterior pituitary hypoplasia, ectopic posterior lobe, and a still visible yet thinner pituitary stalk were included too (see Supplementary Table S6 for further details).

Poorly developed sella turcica has been suggested to be a further characteristic of *LHX4* patients by Tajima et al. in 2013 [39]. However, this trait is inconstant as confirmed by our cases and the literature review: a poorly developed sella turcica was reported in 17/45 patients (37.8%).

Other central nervous system abnormalities have been described occasionally and included pituitary cystic lesions (4/45, 8.9%), persistent craniopharyngeal canal, and corpus callosum hypoplasia.

In *LHX4* carriers, pituitary dysfunction, if present, frequently affects three axes configuring an MPHD. All our probands had at least GH deficiency, and in two of them, a thyrotropin axis deficiency (patient A.III.1 and B.III.3). Patient B.III.2 also presented primary amenorrhea, while her brother, B.III.3, showed a normal pubertal development so far. Gonadotropin axis function assessment was possible only in two of three probands due to the young age of subject B.III.4; however, the absence of detectable estrogen levels during the mini-puberty window suggests hypogonadotropic hypogonadism even in this subject.

Apparently, none of the parents carrying the c. 481C>G in *LHX4* variant, subjects A.II.2 and B.II.11, had pituitary deficits.

Of the 54 patients reported in the literature, a complete pituitary examination was performed only in 46 subjects. Notably, eleven patients (11/46, 15%) presented a normal pituitary function in line with an incomplete penetrance of the genetic trait. Fifty-two percent (24/46) presented at least three pituitary deficiencies; 28% (13/46) had two pituitary hormone deficiencies, and 6% (3/46) presented an isolated deficiency. The most common deficit involved the GH axis, which was present in 86% of the patients, whereas 72% had TSH, 48% ACTH, and 11% PRL deficiency. Of the 20 patients whose pubertal axis could be evaluated, 70% (14/20) presented with FSH/LH deficiencies. Among those with a single-axis impairment, GH was confirmed to be the most frequently affected (please see Supplementary Table S6 for further details).

Notably, in our three probands with the *LHX4* variant, we also detected the co-occurrence of variants in other disease-related genes, *GLI2* and *IGFR1.*

*GLI2* gene encodes one of the three vertebral transcription factors involved in the Sonic Hedgehog pathway, which plays a crucial role in the division of the eye field and brain into 2 halves [40,41,42]. The gene is located on chromosome 2q14 and contains at least 13 exons. The protein encoded with its 1586 amino acid lengths presents a zinc finger DNA binding domain characterized by an N-terminal transcriptional repressive activity and transcriptional activating activity at the carboxyl terminus.

*GLI2* is important for appropriate cell differentiation and pituitary progenitor proliferation [43].

In humans, heterozygous *GLI2* pathogenic variants seem to explain less than 1% of MPHD; however, in some cohorts, the rate rises to 13.6% [44,45]. It causes a wide range of clinical phenotypes, from asymptomatic carriers to Kallman syndrome (MIM147950) and more severe clinical conditions, including Culler–Jones syndrome (MIM 615849) and holoprosencephaly 9 (HPE9, OMIM 610829) showing incomplete penetrance due to complex pattern of inheritance combining multiple environmental and genetic factors, such as variants at other loci or digenic inheritance [45,46].

In this paper, patient A.III.1 was found to also carry the variant of uncertain significance c.2105C>A; p.Pro702Gln inherited from his mother.

Although many studies screening MPHD patients detected *GLI2* more frequently than *LHX4* as the disease-causing gene and despite the low GnomAd frequency (0.00000805), we considered the c.2105C>A not implied in determining the phenotype of the patient, also supported by results of the different in silico predictors used (Supplementary Table S4). Moreover, a brain MRI of the carrier mother did not reveal even minor abnormalities of the hypothalamus and pituitary, and her hormonal function was intact (see Supplementary Materials, Figure S3).

On the other hand, in the affected siblings of family B, an *IGF1R* missense variant (c.166G>A; p.Glu56Lys) was also present, inherited from their father.

The *IGF1R* gene is located on chromosome 15q26.3 and contains 21 exons spanning about 100 kb. The gene encodes 1367 amino acid receptor precursors belonging to the family of tyrosine kinase receptors [47].

In humans, *IGF1R* heterozygous pathogenic variants explain a small percentage of cases with intrauterine growth retardation (IUGR) that remain small because of a lack of catching-up growth [48,49,50]. Nine variants in the *IGF1R* gene have been reported so far [49]. Subsequently, cohort studies also demonstrated heterozygous variants can reduce IGF1 receptor expression through a dominant-negative effect [51,52]. A much more severe phenotype characterized by mental retardation, dysmorphic features, and severe short stature has been described in homozygous patients [53].

Although the identified *IGF1R* missense variant was classified as a VUS, unlike the co-occurring *GLI2* variant in family A, different in silico predictors suggest a possible pathogenic effect (Supplementary Table S4).

Supporting this hypothesis, both probands, B.III.2 and B.III.3, were born SGA and had microcephaly, typical features of IGF1 resistance. They both showed a poor response to the rGH replacement therapy despite a severe GH deficiency in contrast to the patient from family A, who t had the *LHX4* variant. Poor response to rGh is typical of the IGF1R-mutated patients [54,55]. Furthermore, the older brother, subject III.1, carrying only the *IGF1R* variant, was born SGA and presented a final short stature, while the younger sister, subject III.4, carrying only the *LHX4* variant, was born normal for gestational age.

VUS interpretation and report are extremely challenging for geneticists and clinicians. They need to balance the benefit of expanding the knowledge of genetic diseases against the risk of making a spurious association between disease and a poorly characterized but truly benign variant detected on sequencing. Furthermore, they are called to pay special attention when providing counseling to families with VUS results since patients often view genetic results as definitive rather than probabilistic. Defining a VUS as irrelevant or relevant for the phenotype requires the collection of further evidence, with the possibility that a clinician’s judgment on variant pathogenicity may potentially override ACMG classification. For the present study, we considered it useful to also report these two VUS-classified variants in order to (1) alert other clinicians to evaluate VUS thoroughly and carefully by meticulously collecting clinical data and using predictive tools when functional studies are not feasible and (2) to share these rare variants with the scientific community, as “uncertain” does not mean “useless” but may lead to progress in understanding their still unexplored pathogenicity or benignity.

On the other hand, the identification of additional genetic variants through NGS highlights the potential positive impact of such techniques in understanding complex and atypical phenotypes. In two of our *LHX4* cases, in fact, *IGF1R* variant identification answered why the patients presented a poor rGH response, also offering the chance of personalized medicine (in these specific cases, an increase in rGH dosage to achieve a better therapeutic outcome).

This work has some limitations, the main one being the lack of functional studies that could possibly delineate the impact of each variant identified in the patients’ phenotypes, even the VUS in *GLI2*. Secondly, we are not able to investigate a possible founder effect of the novel *LHX4* variant, a hypothesis that remains suggestive.

The main strength of this paper is the thorough characterization of several subjects carrying the same novel *LHX4* variant, describing the endocrinological and neuroradiological features of the carriers.

## 5. Conclusions

We described the clinical and radiological features of three children with MPHD carrying a likely pathogenic variant in *LHX4* combined with a VUS variant in *GLI2* (in subject A.III.1) or *IGF1R* (in subjects B.III.2 and B.III.3).

The co-occurrence of more than one disease-causing gene variant is an emerging issue of exome sequencing applications, leaving the clinician with the challenge of identifying which variant/variants have clinical relevance. A further variant in a second gene somehow involved in growth can explain unusual findings of phenotype, such as the scarce response to GH replacement.

Genetic counseling in such cases can be very challenging due to complex phenotypes and the possible role of oligogenic combinations in the pathogenesis of complex human disorders.

## Figures and Tables

**Table 1:** Clinical, genetic, and radiological findings of probands and their carrier relatives and siblings.

	FAMILY A			FAMILY B					
	II.2	II.4	III.1	II.1	II.11	III.1	III.2	III.3	III.4
**Gender**	M	F	M	M	F	M	F	M	F
**Age at last examination (yrs)**	36.7	31.4	1.8	41.3	36.7	21.0	15.3	4.7	0.4
**Genotype**	*LHX4* c.481C>G	*GLI2* c.2105C>A	*LHX4* c.481C>G; *GLI2* c.2105C>A	*IGF1R* c.166G>A	*LHX4* c.481C>G	*IGF1R* c.166G>A	*LHX4* c.481C>G, *IGF1R* c.166G>A	*LHX4* c.481C>G, *IGF1R* c.166G>A	*LHX4* c.481C>G
**Clinical features**									
SGA birth	-	-	-	-	-	+	+	+	-
Growth failure/short stature	-	-	+	+	-	+	+	+	+
MPHD	-	-	+	N.A.	-	-	+	+	+
Poor rGH response	N.A.	N.A.	-	N.A.	N.A.	N.A.	+	+	N.A.
**Neuroradiological features**									
Posterior pituitary gland ectopy	-	-	+	N.A.	-	N.A.	+	+	+
Small sella turcica	-	-	+	N.A.	-	N.A.	+	+	-
Anterior pituitary hypoplasia	-	-	+	N.A.	-	N.A.	+	+	+
Thinner pituitary stalk	-	-	-	N.A.	-	N.A.	+	+	+
Interrupted pituitary stalk	-	-	+	N.A.	-	N.A.	-	-	-
Minor pituitary abnormalities	+	-	-	-	+	-	-	-	-

Legend: SGA, small for gestational age; MPHD, multiple pituitary hormone deficiency; N.A., not applicable (subject who has not received rGH treatment or/and has not performed brain MRI).

## Data Availability

The datasets used and/or analyzed during the current study are available from the corresponding author upon reasonable request. The data are not publicly available due to privacy reasons.

## Supplementary Materials

The following supporting information can be downloaded at https://www.mdpi.com/article/10.3390/children12030364/s1, Figure S1: Sagittal MR images T1 weighted (A) and T2 weighted (B); Figure S2: Sagittal MRI images T1 weighted (A) and T2 weighted (B); Figure S3: Sagittal MRI images T1 weighted (A) and T1 weighted with gadolinium (B); Table S1: Family A patient III-1 complete hormonal profile; Table S2: Patient III-2 complete hormonal profile at presentation (T0) and after 5.3 years of therapy (T2); Table S3: Patient III-3 complete hormonal profile at presentation (T0) and after 1 year of therapy (T2); Table S4: further gene variants found in probands; Table S5: Family B patient III-4 complete hormonal profile; Table S6: LHX4 gene variants review. Reference [21,23,24,25,27,28,29,30,31,32,33,34,35,36,37,56] has been cited in Supplemental Material Table S6.
