# Supplementary material for: A Novel Missense Variant in LHX4 in Three Children with Multiple Pituitary Hormone Deficiency Belonging to Two Unrelated Families and Contribution of Additional GLI2 and IGFR1 Variant
Source: Children (Basel). 2025 Mar 14;12(3):364. doi: 10.3390/children12030364 (PMC11941417; doi:10.3390/children12030364)
Supplement: Supplementary file 1 [file children-12-00364-s001.zip › children-3478755-SUPPLEMENTAL MATERIAL, TABLES_ revised.pdf]

Supplementary materials

**Hormones**	T0	Reference value
PUBERTAL STAGE	PH1G1	-
GH-peak, ng/mL	1.18	>8
IGF1, ng/mL	7.1	14-203
ACTH, pg/ml	39.32	7.2-63
Cortisol, µg/dl	13.8	2.2-13
PRL, ng/mL	11.97	3-25
TSH, mIU/L	1.15	0.7-6.0
FT4, ng/dl	0.65	1.0-1.8
LH, mUI/ml	0.19	< 1.3
FSH, mUI/ml	0.77	< 1.9
Testosterone, pg/mL	0.03	<10 ng/dL

Table S1- Family A patient III-1 complete hormonal profile

**Hormones**	**T0**	**Reference values**	**T2**	**Reference values**
**PUBERTAL STAGE**	**PH1B1**	**-**	**PH5B5**	**-**
GH-peak, ng/mL	5.8 and 2.7	<8	N.A.	-
IGF1, ng/mL	21.23	51-334	274.7	136-729
Cortisol, µg/dl	7.1	2.2-13	7.18	2.2-13
PRL, ng/mL	5.5	3-25	7.5	4-30
TSH, µIU/ml	3.12	0.6-4.8	2.7	0.5-4.3
FT4, ng/dl	0.98	1.0-1.7	0.8	1.0-1.6
LH, mUI/ml	0.2	< 3.1	9.2	0.5-41.7
FSH, mUI/ml	7	0.6-4.1	7.7	0.3-7.0
17-beta-estradiol, pg/mL	10	<20	18.5	15-85

Table S2- Family B patient III-2 complete hormonal profile at presentation (T0) and after 5.3 years of therapy (T2)

Hormones	T0	Reference values	T2	Reference values
IGF1, ng/mL	6.03	14-203	138.4	16-222
Cortisol, µg/dl	8.3	2.2-13	9.3	2.2-13
PRL, ng/mL	38.7	3-25	24.7	3-25
TSH, µIU/ml	3.8	0.7-5.0	1.2	0.7-5.0
FT4, ng/dl	0.84	1.0-1.8	0.51	1.0-1.8
LH, mUI/ml	0.23	< 1.3	-	
FSH, mUI/ml	0.13	< 1.9	-	
Free-Testosterone	undetectable	<10 ng/dL	-	

Table S3- Family B patient III-3 complete hormonal profile at presentation (T0) and after 1 years of therapy (T2)

Gene (NM)	LHX4 (NM_033343)	IGF1R (NM_000875)	GLI2 (NM_005270)
HGVS cDNA, ref/alt	c.481C>G	c.166G>A	c.2105C>A
HGVS Protein	p.Arg161Gly	p.Glu56Lys	p.Pro702Gln
Exon	4 of 6	2 of 21	13 of 13
CADD_v1.3	24.3	31	18.2
REVEL	0.923	0.815	0.183
AlphaMissense	0.9998	0.9625	0.08539
ACMG/AMP class (criteria)	Likely Pathogenic (PP3: Strong, PM1: Supporting, PM2: Supporting)	VUS (PP3: moderate, PM1: supporting)	VUS (PM1 supporting, BP1 supporting)
Inheritance	P/M	P	M
GnomAD frequency	6.20e-7	Not reported	0.00000805
Homozygous allele number	0	Unknown	0

Table S4. Variants found in genes responsible of congenital hypopituitarism or short stature through WES on trio in our index cases and their interpretation.

Hormones	T0	Reference value
GH-peak, ng/mL	-	
IGF1, ng/mL	22.15	14-203
ACTH, pg/mL	3.8	7.2-63
Cortisol, µg/dl	0.5	2.2-13
PRL, ng/mL	38.7	3-25
TSH, µIU/ml	0.23	0.7-7.4
FT4, ng/dl	0.54	0.9-2.0
LH, mUI/ml	<0.10	< 0.4
FSH, mUI/ml	<0.30	1.2-12.5
17.β-estradiol	undetectable	<20

Table S5- Family B patient III-4 complete hormonal profile

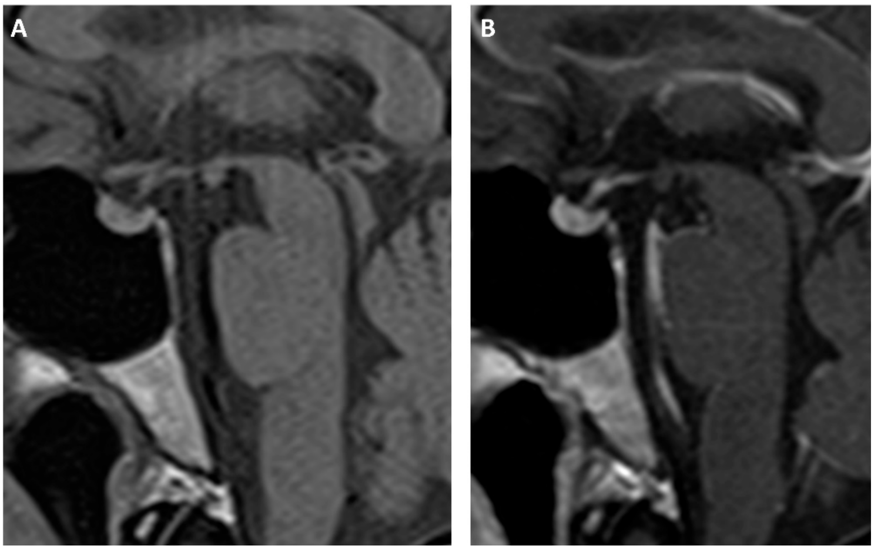

Figure S1. Sagittal MR images T1 weighted (A) and T2 weighted (B)

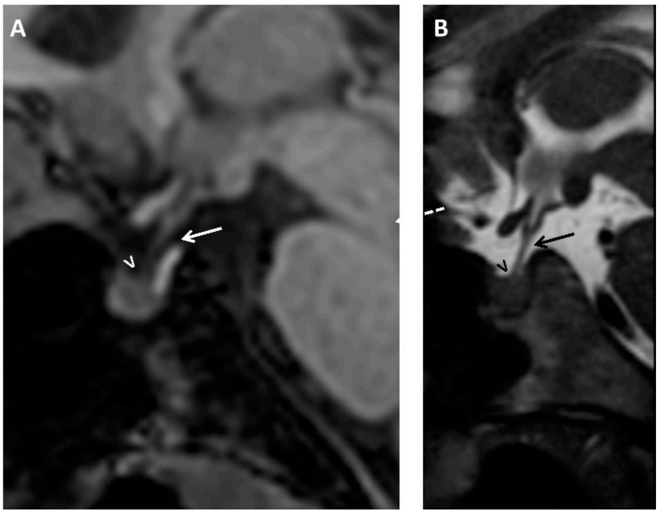

Figure S2. Sagittal MRI images T1 weighted (A) and T2 weighted (B). Regular anterior pituitary lobe (>) located inside small poorly developed sella turcica, with posterior lobe located in the pituitary fossa (arrow).

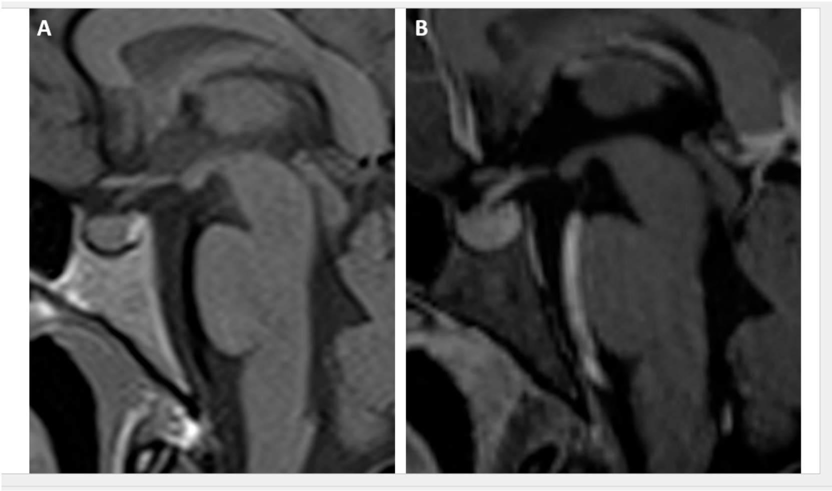

Figure S3. Sagittal MRI images T1 weighted (A) and T1 weighted with gadolinium (B)
