# Supplementary figures and images for: A Novel Missense Variant in LHX4 in Three Children with Multiple Pituitary Hormone Deficiency Belonging to Two Unrelated Families and Contribution of Additional GLI2 and IGFR1 Variant
Source: Children (Basel). 2025 Mar 14;12(3):364. doi: 10.3390/children12030364 (PMC11941417; doi:10.3390/children12030364)

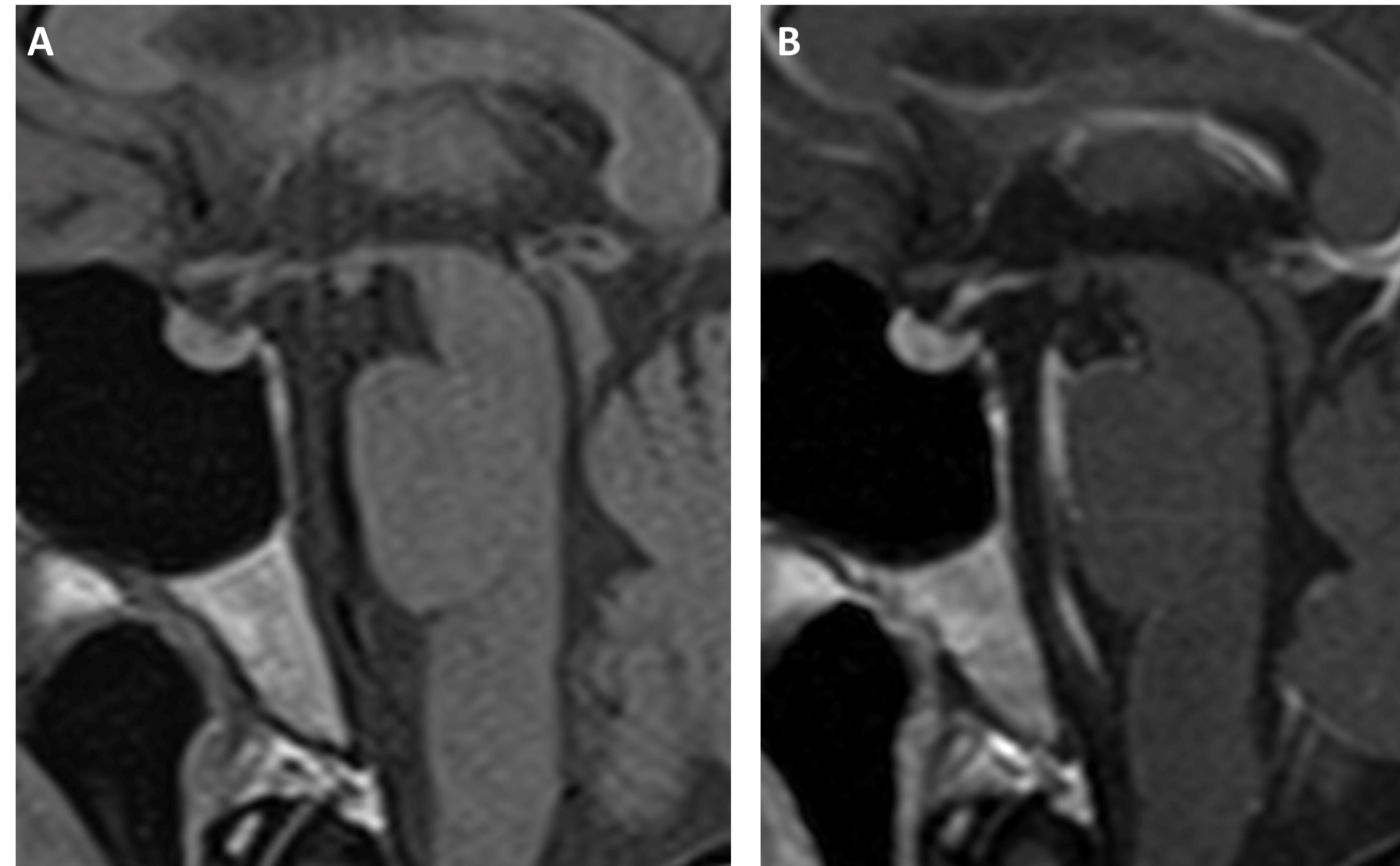

Supplement: Supplementary file 1 [file children-12-00364-s001.zip › children-3478755-FigureS1_LHX4.jpg]

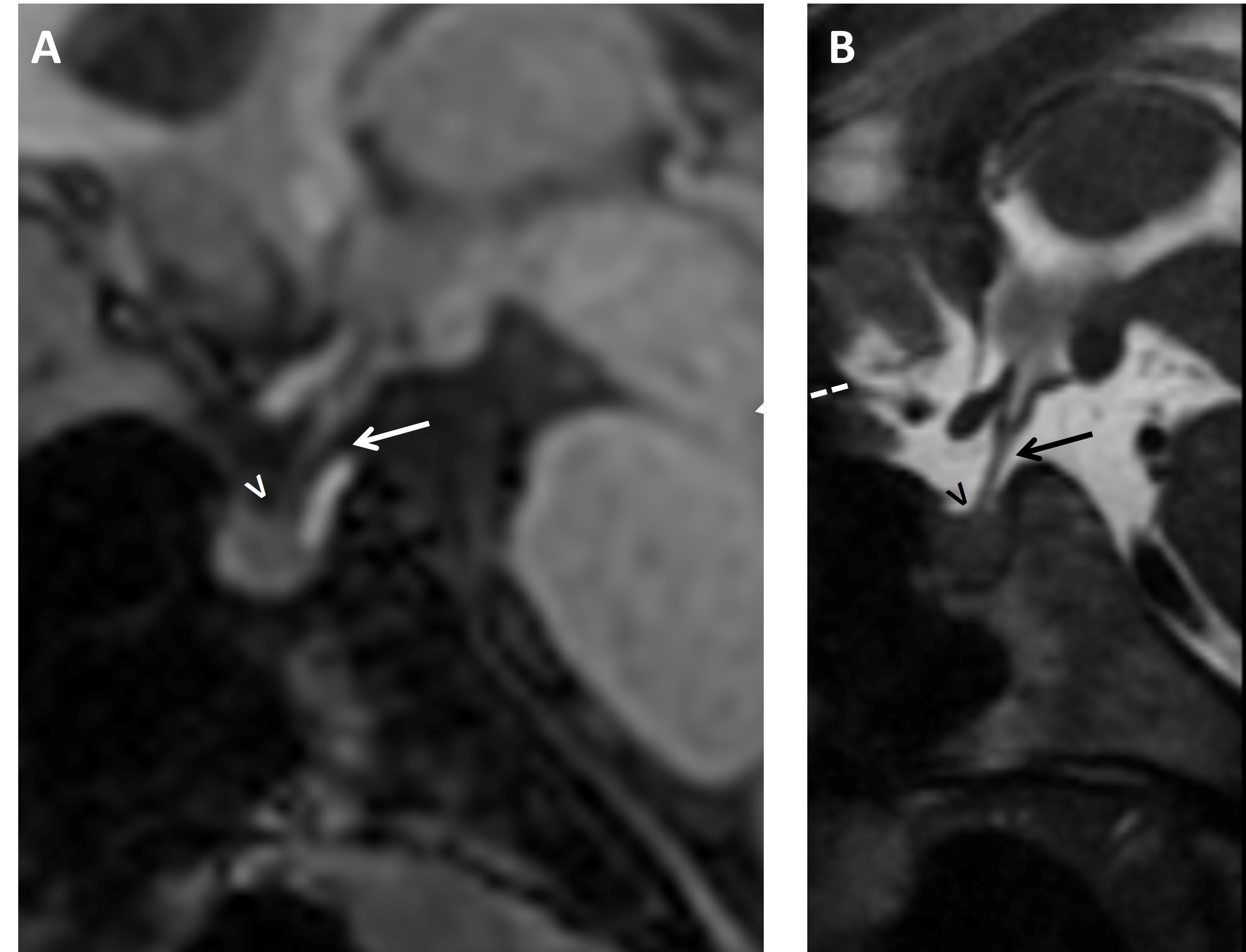

Supplement: Supplementary file 1 [file children-12-00364-s001.zip › children-3478755-figureS2_LHX4.jpg]

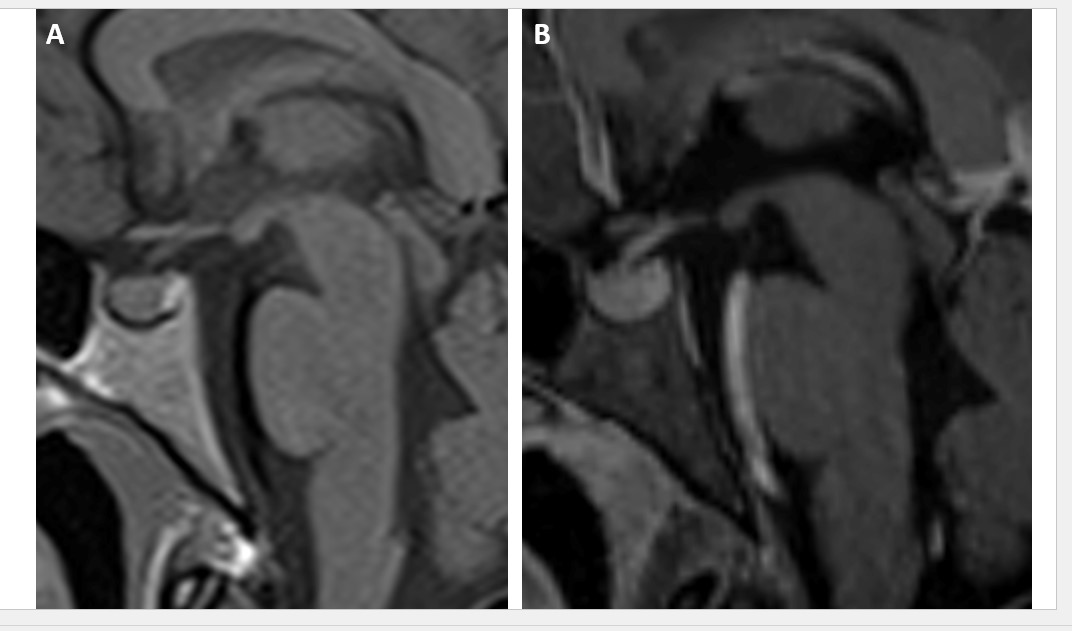

Supplement: Supplementary file 1 [file children-12-00364-s001.zip › children-3478755-figureS3_LHX4.jpg]
